# Microlens Hollow-Core Fiber Probes for Operando Raman Spectroscopy

**DOI:** 10.1021/acsphotonics.4c00525

**Published:** 2024-07-22

**Authors:** Megan J. Groom, Ermanno Miele, Jonathan Pinnell, Matthew G. Ellis, Jessica B. McConnell, Hesham Sakr, Gregory Jasion, Ian Davidson, Natalie Wheeler, Yongmin Jung, Francesco Poletti, Svetlana Menkin, Marlous Kamp, Jeremy J. Baumberg, Tijmen G. Euser

**Affiliations:** † Nanophotonics Centre, Department of Physics, Cavendish Laboratory, University of Cambridge, Cambridge CB3 0HE, U.K.; ‡ The Faraday Institution, Quad One, Harwell Science and Innovation Campus, Didcot, Oxford OX11 0RA, U.K.; § Yusuf Hamid Department of Chemistry, University of Cambridge, Cambridge CB2 1EW, U.K.; ∥ Optoelectronics Research Centre, University of Southampton, Southampton SO17 1BJ, U.K.; ⊥ Van ‘t Hoff Laboratory for Physical & Colloid Chemistry, Department of Chemistry, Utrecht University, Utrecht 3584 CH, The Netherlands

**Keywords:** hollow-core fiber, Raman, microlens, in situ, fiber probe, photonic nanojet

## Abstract

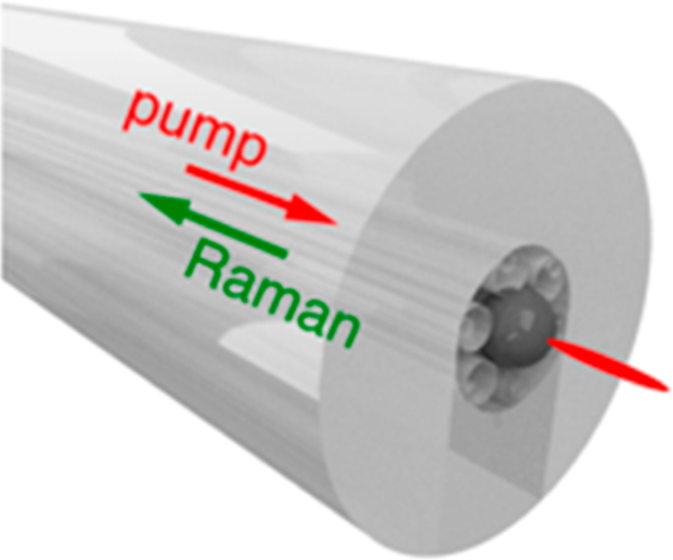

We introduce a flexible microscale all-fiber-optic Raman probe which can be embedded into devices to enable operando in situ spectroscopy. The facile-constructed probe is composed of a nested antiresonant nodeless hollow-core fiber combined with an integrated high refractive index barium titanate microlens. Pump laser 785 nm excitation and near-infrared collection are independently characterized, demonstrating an excitation spot of full-width-half-maximum 1.1 μm. Since this is much smaller than the effective collection area, it has the greatest influence on the collected Raman scattering. Our characterization scheme provides a suitable protocol for testing the efficacy of these fiber probes using various combinations of fiber types and microspheres. Raman measurements on a surface-enhanced Raman spectroscopy sample and a copper battery electrode demonstrate the viability of the fiber probe as an alternative to bulk optic Raman microscopes, giving comparable collection to a 10 objective, thus paving the way for operando Raman studies in applications such as lithium battery monitoring.

## Introduction

Greater energy storage, computing power, and medical diagnostics are required to meet multiple global challenges. In many such applications, high-performance, safe batteries are crucial. To achieve this, the electrochemical processes that plague current batteries must be identified in detail.

Raman spectroscopy can reveal molecular fingerprints without perturbing a system. Therefore, operando and in situ Raman spectroscopy are promising tools for monitoring the evolving state of batteries. However, a major challenge is that spontaneous Raman signals are inherently weak, and experiments must be carefully designed to achieve the desired sensitivity. This issue can partially be overcome by efficiently delivering and collecting light or by enhancing the sample Raman response. Despite this challenge, operando Raman experiments have already been instrumental in battery science,^1,2^ materials discovery,^3,4^ bioanalytics,^5^ and medical diagnosis.^6,7^ In battery research, operando Raman spectroscopy can help identify electrochemical pathways and degradation processes. Traditionally, such experiments are performed in specialized Raman cells with optical access windows.^8^ A major drawback of such geometries is that the battery’s electrolyte and electrode surfaces significantly differ from those of real devices.

Raman signals can be substantially improved by surface-enhanced Raman spectroscopy using plasmonic particles.^9^ While this method is successfully applied across many sensing applications, introducing additional metallic substrates in batteries significantly perturbs their electrochemistry, limiting their usefulness in operando experiments. Such perturbations can be avoided by electrically isolating the plasmonic nanoparticles with a thin silica layer. Such shell-isolated nanoparticles for enhanced Raman spectroscopy have been used to study battery electrode surface reactions.^10−12^ However, optical access is still provided through specialized Raman cells, hence, limiting the relevance of such measurements on real devices.

A true operando Raman technique for battery science would allow for nonperturbing measurement under realistic cell configurations, with low electrolyte excess and without optical windows, while providing a sufficiently strong optical signal and signal-to-noise ratio. Optical fibers can provide such noninvasive optical access and have recently become an established tool for operando sensing in batteries.^2^ Fiber-sensors have been used to monitor temperature, pressure, and strain with fiber Bragg gratings;^13−16^ states of electrode charge via fiber evanescent wave spectroscopy;^17−20^ and ion distribution through Raman spectroscopy.^21−23^ In all these examples, solid-core fibers have been used, in which light is guided through a glass waveguide core via total internal reflection. For Raman spectroscopy this however leads to unwanted dominant silica background signals, and a second fiber is often used for signal collection to reduce this, adding significant complexity to the sensor.^21,23^

Hollow-core fibers (HCFs) can effectively minimize the inherent silica-glass background signal, allowing excitation light and Raman signals to propagate in the same fiber, giving a significant advantage compared to standard solid-core fibers. Using their different waveguiding mechanism compared to traditional solid-core fibers, HCFs typically comprise a hollow waveguide core surrounded by thin-walled glass structures, allowing light within a certain wavelength range to be guided along the fiber. This was first realized via band gap reflections from photonic crystal glass microstructures.^24,25^ Subsequently, antiresonance reflection fibers have been developed with an apparently simpler internal microstructure,^26−28^ but one which can result in optical losses beyond what is possible in solid core fibers.^29^ Moreover, HCFs can be filled with low-index fluids while still effectively guiding light, making them an excellent choice for near-background-free Raman spectroscopy.^30−33^ For Raman experiments in fluid-filled HCFs, pump light remains focused over an extended path length, enhancing the Raman signal compared to unfilled or solid-core fibers immersed in the same fluid.^34,35^ This method is thus known as fiber-enhanced Raman spectroscopy (FERS).

Recently, Miele et al. used FERS for operando Raman spectroscopy within a working Li-ion pouch cell.^32^ A thin, flexible HCF was embedded inside a commercially relevant pouch cell without significantly altering the cell design or operation. Periodically sampling 24 μL of electrolyte from the cell into the HCF revealed new electrochemical processes occurring during multiple battery cycles. One drawback of the method is that it requires specialized microfluidic controllers to flow electrolytes into and out of the HCF. A more straightforward system would involve a static probe, with no liquid extraction needed. Such a probe would track local chemical changes rather than obtain global measurements. This is particularly desirable since local electrochemical environments seem to vary widely across electrode surfaces during cycling.^23^

Here, we focus on proposing and optically characterizing a static operando HCF Raman probe designed to continuously monitor the Raman spectrum at a fixed position within a sealed system.

A key challenge of using a HCF is that their unique fiber design and operating principle mean HCFs typically have a low numerical aperture (NA), resulting in inefficient coupling and collection of light to/from the fiber core. While this is an active area of research within the area of HCF fabrication, at present, HCFs with a higher NA are not widely available, necessitating the exploration of alternative solutions. One approach to improving the signal is to surround the HCF with high-NA multimode fibers to collect more Raman-scattered light.^36,37^ Unfortunately, this increases the overall diameter of the sensor, making it incompatible with the closely spaced electrodes within Li-ion batteries.

An alternative approach is to use a microlens at the fiber facet, as has been utilized in endoscopic Raman applications.^38−43^ Microlenses are small dielectric spheres whose focusing properties can be controlled by adjusting their size and refractive index.^39^ If the diameter of the microsphere is of the same order of magnitude as the wavelength of the incident light, the microsphere tightly focuses light into a “photonic nanojet” (PNJ).^44−48^ This phenomenon enhances the Raman scattering from a small, well-defined volume of ∼10 μm^3^. In the case of existing microlens-assisted fiber probes, the backscattered Raman signal can be collected by either the same microlens or a surrounding concentric waveguide. This HCF-coupled microlens approach has been applied by Lombardini et al., who developed a sophisticated design where a 30 μm silica microlens was spliced into the core of a 327 μm wide double-clad Kagomé HCF.^40^ A sub-micrometer spot size was achieved, with an estimated NA of 0.5. The probe was then embedded in a 4.2 mm wide metal tube with piezo-scanning elements for endoscopic Raman imaging applications.^40^ Kudlinski et al. extended this work, using a double-clad simplified antiresonant fiber instead.^41^ Existing work on microsphere-assisted fiber-Raman imaging shows the potential success of these systems.^37,40,41^

Our approach differs from earlier work in three ways: first, a high-refractive index microsphere is used to tightly focus and collect light just outside the surface of the microsphere. This configuration is ideal for the proposed nonscanning single-point Raman measurements on battery electrodes, as opposed to earlier scanning endoscopy work that used lower-index spheres. Second, a nested antiresonant fiber (NANF) is used, offering a simpler fiber design compared to kagomé-type HCFs used in previous work. Important features of NANFs are low transmission losses, robust mode guidance, and a compliant glass structure that facilitates probe fabrication. Furthermore, they offer the potential to significantly reduce the overall probe diameter, which is essential in confined spaces within batteries. Third, we present a novel experimental method to independently characterize both the excitation and collection volumes of the probe. The approach uses a single-mode fiber coated by a thin layer of CdTe quantum dots (QDs) as an IR isotropic point source.

We thus developed and characterized an HCF-microlens Raman probe that is as small and simple as possible and suitable for reactive and confined environments. The relatively simple construction and relatively small outer diameter allow it to be embedded in highly confined spaces, such as Li-ion batteries. The overall probe diameter is minimized by using a nested antiresonant nodeless HCF integrated with a high-refractive index microsphere, unifying excitation, and collection.

The excitation profile and numerical aperture of the fiber probe are characterized by scanning optical microscopy and compared to simulations. The collection efficiency of the probe is tested using an isotropic point light source comprising a single-mode fiber whose core is covered by a layer of IR-emitting colloidal QDs. The device performance is demonstrated on a Raman sample with relatively high scattering angles. Finally, the device is used for ex situ proof-of-concept measurements on lithium anode-free battery electrodes (with a copper current collector).

## Principle

### Nested Antiresonant Nodeless HCF

The hollow core fiber used in this work is a NANF.^28,29,49^ The guidance properties of this type of HCF are consistent with the antiresonant reflection waveguide (ARROW) model, which calculates resonant wavelengths for which light can resonantly leak out of the fiber through the glass struts.^26^ At wavelengths within transmission windows between these resonances, the cladding layer acts as a mirror, and light is guided along the fiber. NANFs have recently achieved record low transmission losses compared to other types of fiber.^28,50,51^

Due to their simpler internal structure, NANF allows for a thinner fiber diameter compared to, for example, bandgap or kagomé fibers, which is important for insertion into small sample spaces. While the outer diameter of the NANF used in this work is 200 μm, smaller outer diameters can easily be achieved by reducing the thickness of the solid glass cladding, without a loss in performance. The innovation of the NANF design, compared to other simplified tubular fibers, makes its guidance less sensitive to the temperature and pressure changes that usually occur during battery cycling.

### Constructing the Fiber–Microlens Probe

**Figure 1:**
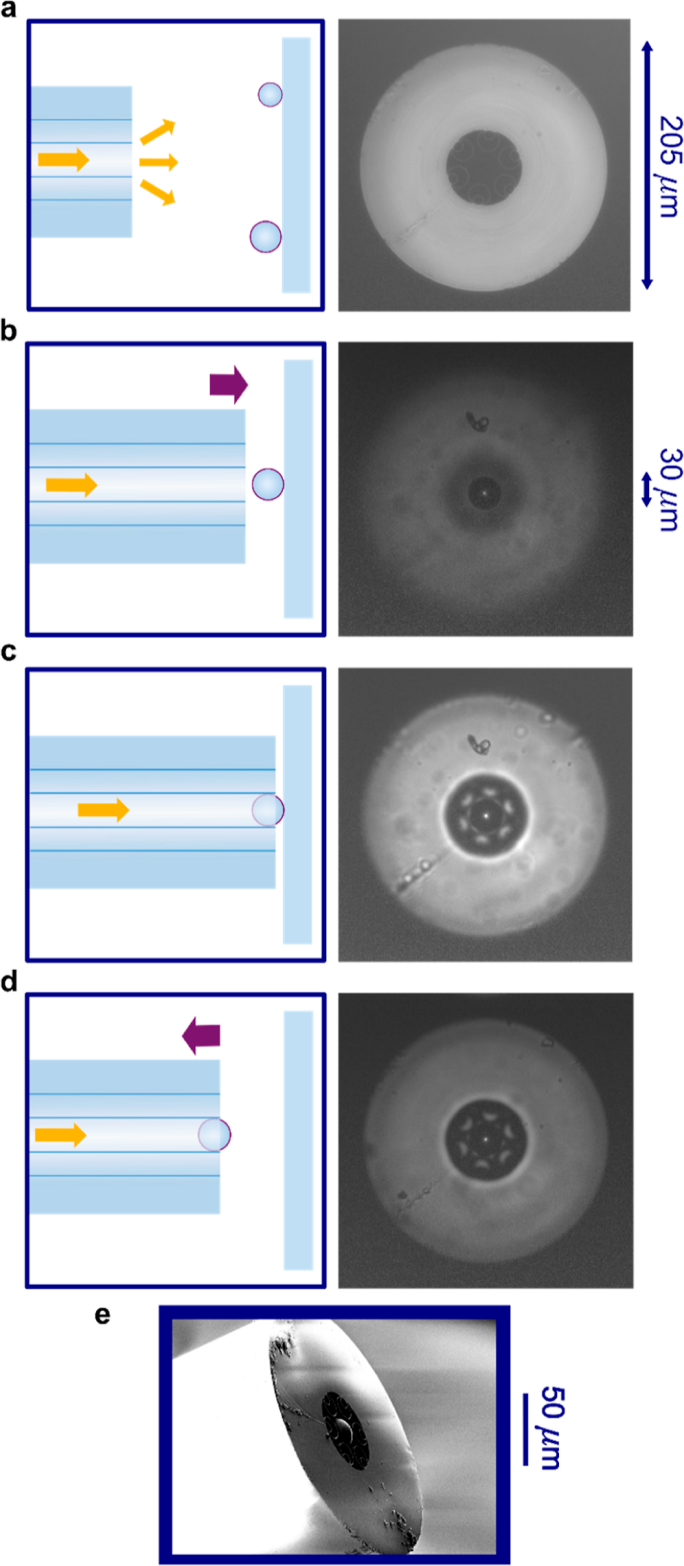
Fiber–microlens manufacturing protocol. Schematics and white light images depict each stage of embedding the microlens into the HCF. (a) HCF imaged through a glass slide. (b) Microlens on glass slide, with fiber facet aligned beyond the focus. (c) Fiber facet in contact with microlens on a glass slide. (d) Microlens embedded inside hollow core of the fiber facet. (e) Angled scanning electron microscopy (SEM) image of fiber–microlens.

The probe is constructed by dispersing high-refractive index microspheres onto a glass slide and imaging the freshly cleaved fiber facet from the backside (Figure 1a). Using translation stages to align an appropriately sized sphere with the NANF core (Figure 1b), the fiber facet and sphere are brought into contact (Figure 1c). The internal structure of the NANF allowed for embedding microlenses securely without any additional postprocessing steps. The slight elastic deformation of the microstructured glass accommodates the microlens (Figure 1d,e). The microlens can therefore be embedded in the fiber core without complex fiber postprocessing.

## Results and Discussion

### Optical Transmission of the NANF Probe

**Figure 2:**
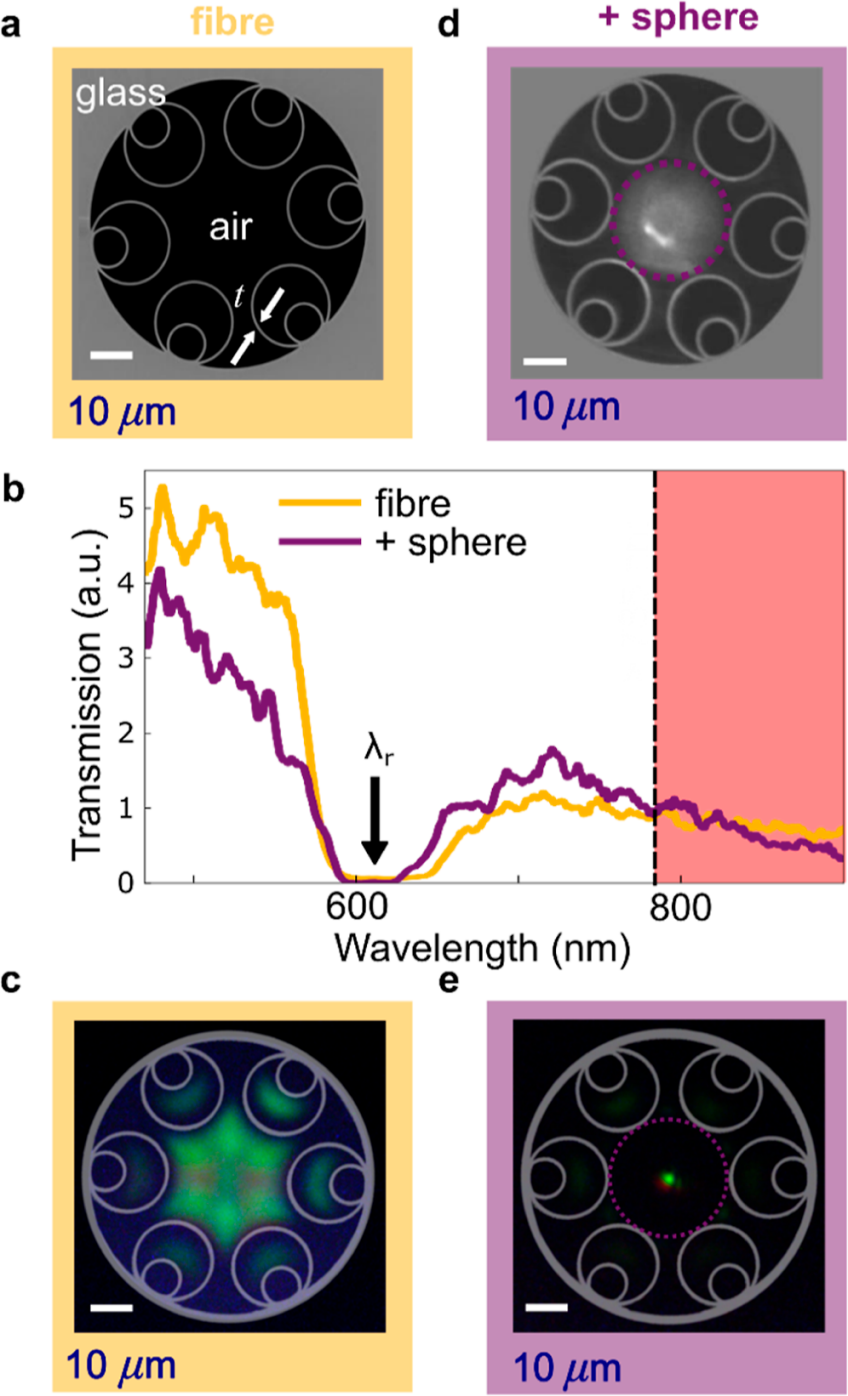
Imaging and transmission of NANF and fiber-probe. (a) SEM images of an unmodified NANF facet. (b) Transmission of NANF with and without microlens. (c) Image of NANF facet, with a super continuum laser coupled in. (d) SEM image of the fiber-microlens probe. (e) Image of a fiber–microlens probe with super continuum laser coupled in.

The HCF used has an antiresonance window ideally placed for Raman spectroscopy at 785 nm and was fabricated and previously characterized by Sakr et al.^29^ The fiber has a relatively small outer diameter of 205 μm (Figure 1a). Its internal hollow structure is 65 μm wide, comprising a central core surrounded by six glass tubes, each containing a smaller-diameter glass tube to reduce optical losses (Figure 2a). The inner core has an internal diameter of ∼28 μm, and further details of the internal geometry can be found in Supporting Information Figure S1 and Table S1. The glass strut thickness, *t*, of 580 nm results in a resonance, λ_r_, at 620 nm (Figures 2b and S2). Sakr et al. confirm that the first resonance is at 1021 nm, corresponding to a Raman shift of 2945 cm^–1^ (Figure S3).^29^ Hence, the antiresonance window of this NANF is wide enough to monitor the entire fingerprint region of the Raman spectrum.

The NANF fiber has a strong antiresonance around 500 nm (Figure 2b), which can be observed when light from a broadband supercontinuum source is coupled into the fiber, launching a superposition of waveguide modes with a dominant contribution in the green wavelength range (Figure 2c).

The fiber probe is constructed by embedding a barium-titanate glass sphere into the hollow core of the NANF. Fiber lengths between 6 and 8 cm were used. This “microlens” with a diameter of ∼28 μm fits tightly and has a reported refractive index of 1.9. It is clamped by the slightly compliant 580 nm thick glass struts (Figure 2d). When light is launched into the unmodified facet (Figure 2c), it is tightly focused as it passes through the microlens (Figure 2e).

The NANF fiber guides light at the 785 nm pump wavelength and Raman shifts of up to 2945 cm^–1^. Figure 2b shows that adding the high refractive index microlens does not affect the transmission window of the fiber in the Raman region of interest, confirming its suitability for broadband Raman measurements.

### Optical Characterization of Excitation

The exact intensity distribution and NA of the focused 785 nm pump light are essential for evaluating the probe performance in Raman measurements. The intensity distribution close to the surface of the microlens is therefore reconstructed by scanning different positions along the axis of the probe and acquiring an image at each position.

### Scanning Optical Microscopy

**Figure 3:**
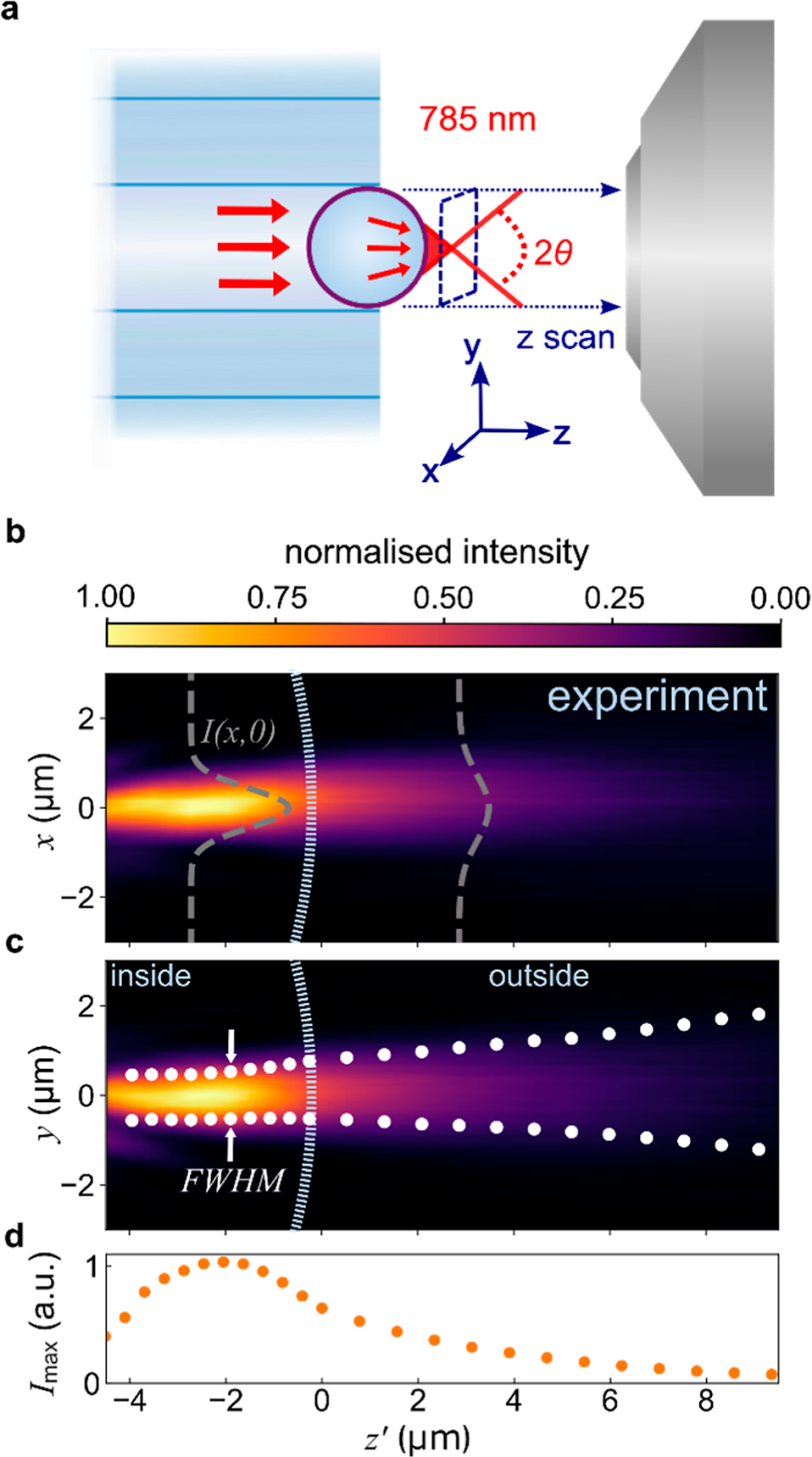
A scanning optical microscopy experiment (a) shown schematically. (b) Reconstruction of the excitation electric field intensity in the *xz* plane, showing the Gaussian distribution fitted to the *xy* intensity at each *z*′ position. (c) Reconstruction of the excitation field intensity in the *yz* plane, showing the fitted Gaussian at each *z*′ position. (d) Measured peak intensity at the center of the Gaussian fit (*x*_0_,*y*_0_) at each *z*′ position.

785 nm light is coupled into the unmodified input facet using a 10× microscope objective. Once a symmetric and sufficiently intense mode exiting the sphere is achieved by optimizing the in-coupling, the relative position between the in-coupling objective and probe is fixed. In the present case, the most qualitatively symmetrical mode is deemed optimal rather than the most intense transmission. For collection, a 60× microscope objective is placed on a computerized translation stage, which moves the objective along the *z* direction relative to the probe (Figure 3a), similar to Ferrand et al.^52^ To provide a realistic insight into the intensity distribution imaged inside the dielectric material, *z* is adjusted for the changing refractive index along the *z*-axis: the corrected coordinate *z*′ is defined as *z*′ = *z*/1.9 for *z* < 0 and *z*′ = *z* elsewhere. At each *z*′ position, a 2-dimensional image in *x* and *y* is captured. The sequence of optical slices reconstructs the output field of the fiber–microlens probe. The cross-section of each image can be taken through the mode center (*x*_0_,*y*_0_) along either the *x* or *y* axes and projected along the *z* axis (Figure 3b,c), showing the shape of the excitation beam focused by the microlens.

### Analysis of Scanning Optical Microscopy

At each *z*′ position, a circular Gaussian is fit to the two-dimensional image, , with amplitude *A*, full-width-half-maximum , and center  = (*x*_0_,*y*_0_). The change of fwhm with *z*′ is used to estimate the angular divergence θ of the focused excitation beam. This angular divergence is measured from the sphere surface (last point of light refraction), giving the numerical aperture NA = *n* sin θ (with *n* = 1 in air).

### Direct Imaging of Focused Light

The intensity distribution remains narrow perpendicular to the direction of light propagation (*x*,*y*) for propagation distances of several μm (Figure 3b,c). This resembles a PNJ, although these are normally obtained for microlenses closer to wavelength-scale dimensions.^48^ As expected from the symmetry of the HCF and the self-aligned axial registration of the microlens, the intensity distribution is near-symmetrical in *x*,*y*, giving a circular rather than elliptical Gaussian fits. We note that the narrowest part of the beam (and thus the maximum intensity, Figure 3d) lies inside the sphere for the *n* = 1.9 barium titanate glass. Outside the sphere, the smallest fwhm (1.1 μm) occurs at the sphere surface, with an NA of 0.12 (Figure 3c).

**Figure 4:**
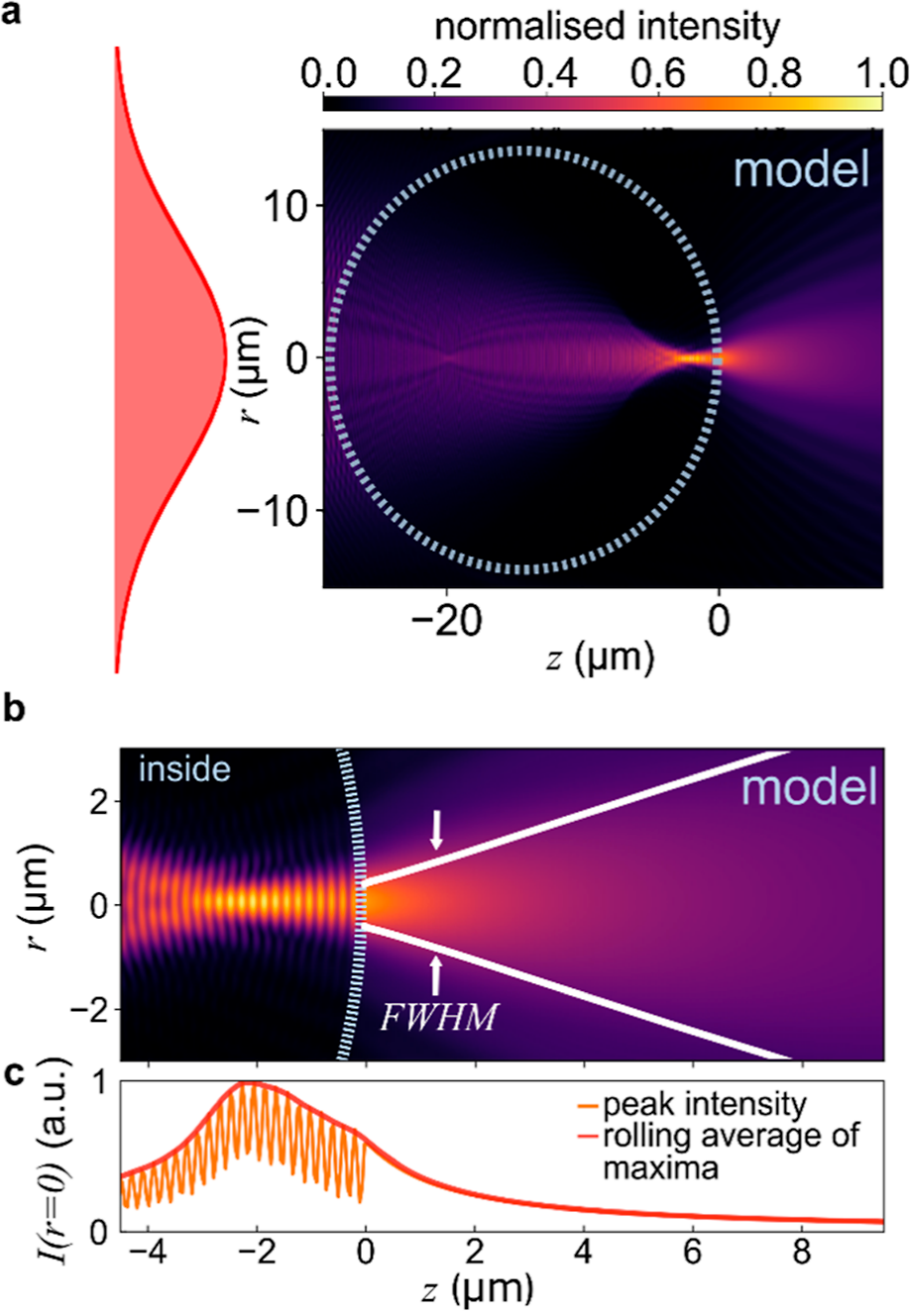
Simulated probe excitation field distribution (a) for a Gaussian input field and a 28 μm microlens. The region of interest from the experiment (Figure 3), showing (b) full-width-half-maximum (fwhm) and (c) peak intensity for the Gaussian fitted at each *z* position for the simulated data, and a rolling average of local maxima.

An effective excitation volume can be defined as being bound by the beam width (over which the intensity falls by a factor of ) and the decay length. The decay length is defined as the distance between the sphere surface and where the excitation and collection signal drop by . The beam diameter expands from 1.9 to 3.8 μm and decays over 4.7 μm, resulting in a cone of effective excitation with a volume of ∼50 μm^3^. Simulated output field from the microlens finite-difference time-domain simulations are carried out to compare the experimental results with theory (Figure 4). These are based on a Gaussian input field, appropriately matching the theoretically obtained fundamental mode of the HCF with fwhm of 16.4 μm, and a spherical microlens with a diameter of 28 μm and refractive index of 1.9 (Figure 4a).

As in the experimental analysis, a Gaussian cross-section is fit to the simulated intensity distribution at each *z* position. The fwhm of the fitted Gaussian plotted against *z* (Figure 4b), shows the optical propagation at 785 nm, as well as the average peak field intensity (Figure 4c). Note that the adjusted *z*′ in Figure 3 is equivalent to the *z* in Figure 4. The narrowest fwhm is inside the microlens, at the position of greatest intensity 2.2 μm inside the sphere surface. Outside the sphere, the narrowest fwhm of 0.91 μm is at the sphere surface, then expanding with an NA of 0.61.

### Comparison of Theoretical Assumptions and Experimental Results

Both experiment and theory confirm that for the barium titanate glass microlens, the focus is a few micrometers inside the sphere. Strikingly, the theoretical intensity diverges five times faster than that observed. The model also predicts a modulated intensity distribution inside the microlens, which is not observed in the experimental results, coming from the interference of light back-reflected from the lens surface and forward-propagating refracted light. This is not observed because the images capture only forward-propagating light.

Given that the beam divergence of the model and experiment are significantly different, we re-examine the model assumptions. Literature suggests that an inhomogeneous microlens refractive index can give less focused and more weakly diverging beams.^53,54^ We therefore surmise that the barium titanate glass spheres do not have a homogeneous refractive index, resulting from their growth evolution.

Having characterized how incident 785 nm light is focused by the fiber-probe onto a Raman sample, with the highest intensity at the microsphere surface and fwhm of 1.13 μm, we now characterize the collection of Raman scattered light by the fiber-probe.

### Characterizing Signal Collection

To characterize the collection efficiency of the probe at different emitter positions, a bright isotropic point source would be ideal. Here, the photoluminescence of QDs was selected to resemble a point source. We select CdTe carboxylic-acid-capped core–shell QDs that absorb at 405 nm and emit at 780 nm. To ensure well-defined spatial uniformity (rather than low intensities from a single QD), an area of many close-packed QDs arranged in a monolayer is excited with a spatially well-defined light source. A solid-core fiber with a single-mode fwhm of 3.8 ± 0.6 μm at 405 nm delivers UV pump light to a layer of QDs coated on its facet. The final sample resembles a Raman scattering point source using this QD layer back-excited by a Gaussian spot of 405 nm light.

### Spectral Mapping of Single-Mode Fiber Output

**Figure 5:**
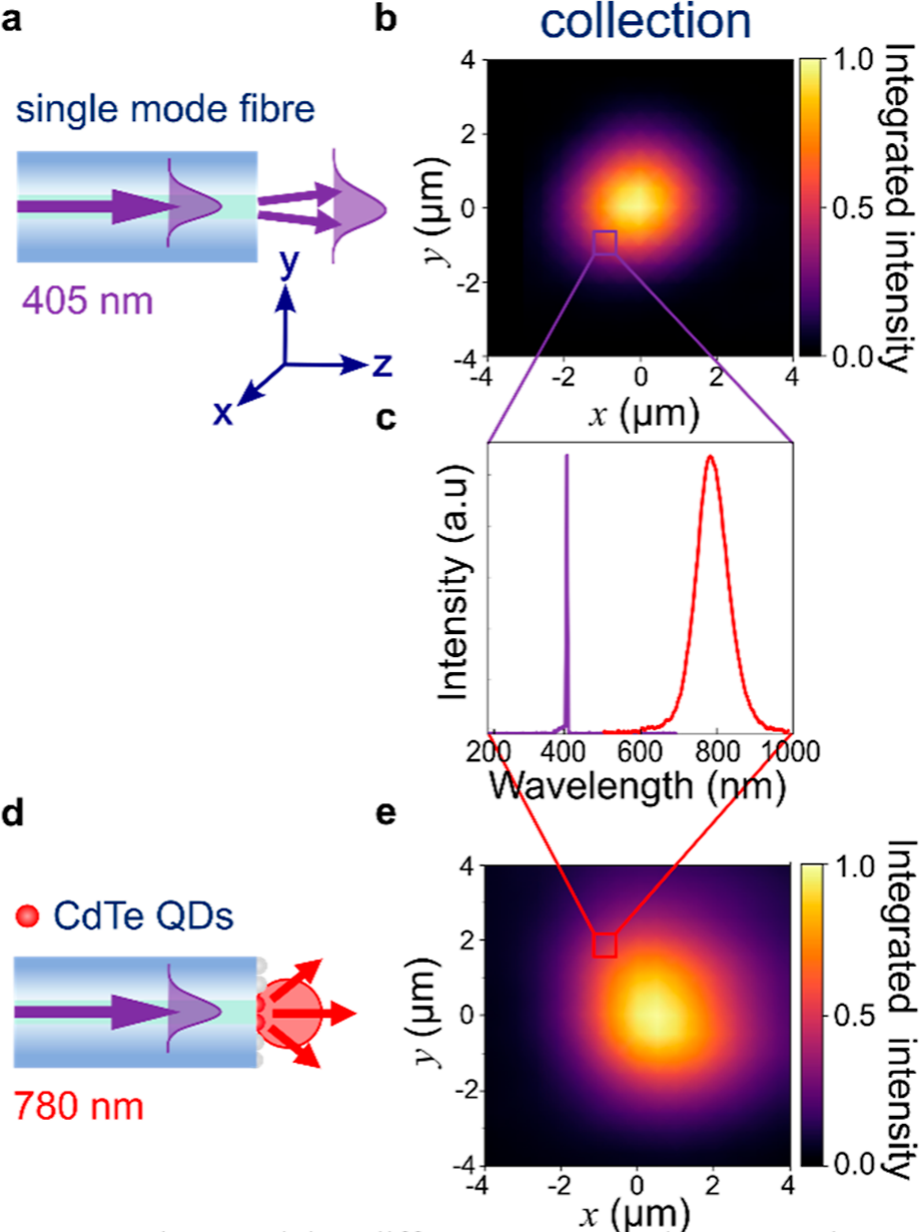
Characterizing different sources using spectral mapping. (a) Schematic of a bare SMF imaged with a 60× objective and (b) spectral map of the 405 nm mode exciting the SMF. (c) Single spectrum from the spectral map of (a,d). (d) Schematic of SMF coated with CdTe QDs, imaged with 60× objective, and (e) spectral map of its emission at 780 nm.

To confirm that the UV light exiting the fiber is spatially well-defined, it is spectrally mapped. A 405 nm laser diode couples light into the SMF with a 4× objective, and a 60× objective collects the transmitted light from the distal end of the SMF into a fiber-coupled spectrometer (Figures S4 and 5a). This distal fiber end is moved in *x*,*y* by a computerized stage relative to the fixed position of the 60× objective, and a single spectrum taken at each position over a 64 μm^2^ area. In the resulting intensity map (Figure 5b), only the single peak at 405 nm is present at each point (Figure 5c). The map has been normalized to the most intense pixel, and Gouraud shading is applied. This map shows the shape and size of the output mode of the SMF, which gives a measured fwhm of 2.7 μm averaged over *x* and *y*, somewhat smaller than the manufacturer specification (fwhm 3.8 ± 0.6 μm).

### Spectral Mapping of Single-Mode Fiber Coated in QDs

Using the identical setup to confirm the shape and size of the light leaving the SMF, we investigated the emission profile of a QD layer on the SMF facet. Their deposition (see Methods) attaches QDs via electrostatic interactions to a monolayer of APTES molecules on the flat glass fiber facet.^55^ This deposition technique is designed to form a single monolayer of QDs and has been checked elsewhere.^55,56^

The CdTe QDs are back-illuminated by 405 nm light, eliciting photoluminescence at 780 nm (Figures 5c and S4) in all directions (Figure 5d). Spectra across the 64 μm^2^ area (Figure 5e) show the fwhm of QD emission is 3.8 μm when back-illuminated by the UV fiber mode. While the spot size of the 780 nm QD emission is slightly larger than the illuminating 405 nm fiber mode, this source is sufficiently small to act as an isotropic point source to characterize the Raman probe.

### Spectral Mapping of Single-Mode Fiber Coated in QDs with a Fiber Probe

**Figure 6:**
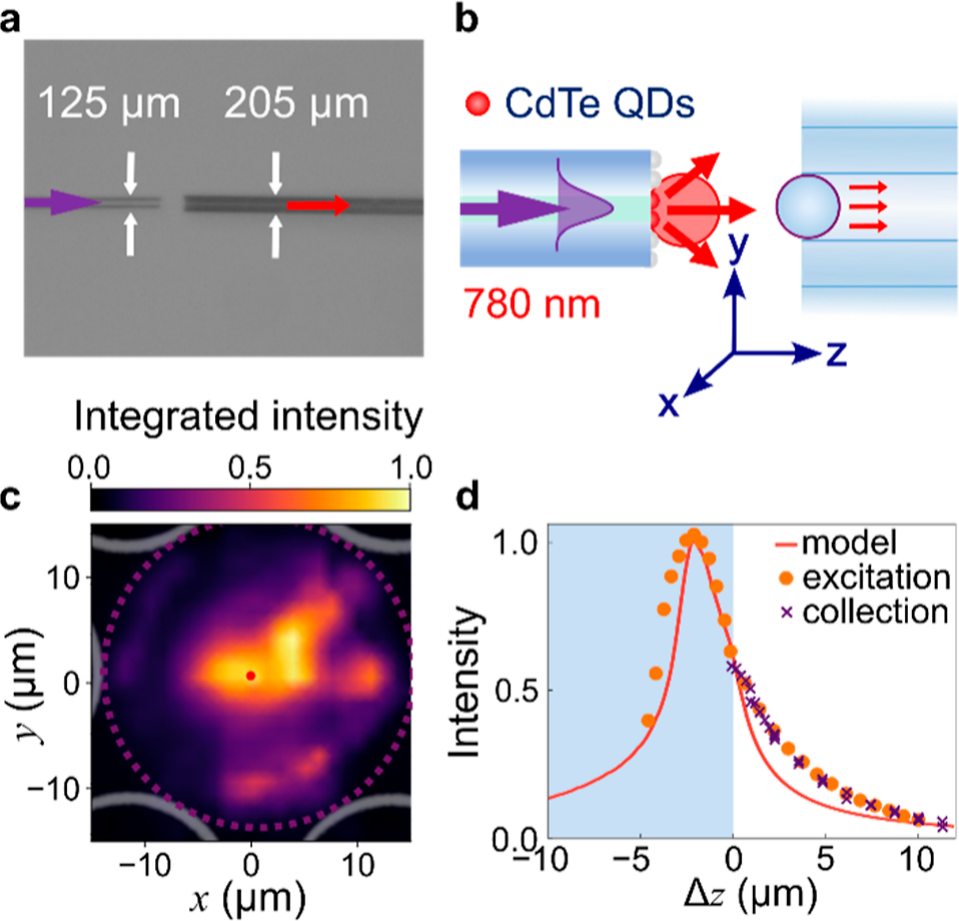
Collection efficiency of the fiber–microlens probe is characterized by measuring QD emission with the probe, shown (a) in side-view and (b) schematically. (c) Probe collection over a spectral 2D *xy* map, with fwhm of the excitation spot (red circle) and microlens diameter (purple dashed). (d) Intensity of a s780 nm luminescence signal at fixed (*x*,*y* vs *z*), compared to simulated excitation and experimental data (Figures 3 and 4).

To characterize its light collection, the 780 nm emission from the QD-coated single-mode fiber is mapped using the microlens probe in place of a microscope objective (Figures 6a,b, and S4). The microlens was placed as close as possible without touching the sample. Spectral maps with 900 spectra cover a 1600 μm^2^ area, where we examine the central 30 × 30 μm, which is treated with Gouraud shading (Figure 6c). The apparent size of QD emission given by the fiber–microlens probe is much larger than that seen by the 60× objective (Figure 5e). It is also much larger than the area excited by the fiber probe (fwhm from Figure 3, shown as a red spot for comparison). The image shows a central collection cone in the inner fiber core, and an additional narrow ring surrounding this. Overlaying the microlens diameter (purple dashed) and HCF facet (gray) shows the relative size of the signal collection area. We note that the narrow ring is of a similar diameter to the microlens and internal diameter of the HCF.

It is apparent that, unlike the spectral map taken with a 60× objective (Figure 5e), the collection shape of the microlens/fiber is not a symmetrical circle. This map is a convolution between the QD source and the point-spread function (PSF) of the probe. Hence, this suggests the probe PSF is not well described by a Gaussian function. One possibility is that the sphere is not entirely spherical, which may be more pronounced in collection compared to excitation as a wider surface region is involved. Another factor that could impact the PSF shape is the uneven excitation of higher-order modes in the NANF. The QD sample emits at a wide range of angles and can thus excite these higher-order modes. If the sphere is not positioned exactly centrally in the fiber core, higher-order modes may be excited unevenly, resulting in the asymmetric PSF observed here.

### Intensity from the QD-Coated Single-Mode Fiber Scanning Fiber–Microlens Probe in *z*

To compare the probe collection cone with the excitation experiment and modeling, the QD-coated SMF is moved in the *z* direction toward the microlens probe. Figure 6d displays the integrated intensity of 780 nm light at a single *xy* position vs the *z* separation from the probe. Peak intensities from the optical scanning microscopy experiment (Figure 3d) and the corresponding simulated peak intensities (Figure 4c) are plotted on the same graph (after background subtraction and scaling).

The position of the sphere surface is found experimentally when the output mode and collected intensity no longer vary, meaning the sphere and sample are in contact (*z* = 0). The SMF and microsphere surface came into contact before the intensity reached a maximum. This confirms that the point of optimal collection is inside the sphere, consistent with our excitation experiments, which found the same.

### Comparing Excitation and Collection

The excitation and collection volumes of the probe were measured independently. By comparing the data in Figures 3 and 6, it appears that the excitation spot size is much smaller than the collection area, which thus defines the lateral sensitivity of the probe in *x* and *y*. Considering the depth sensitivity (Figure 6d), the excitation and collection efficiencies outside the spheres have decay lengths of 4.68 and 4.32 μm, respectively. Thus, the excitation and collection are similarly sensitive to the *z*-position of the sample.

### Raman Spectroscopy

#### Comparison with a 10× Objective

**Figure 7:**
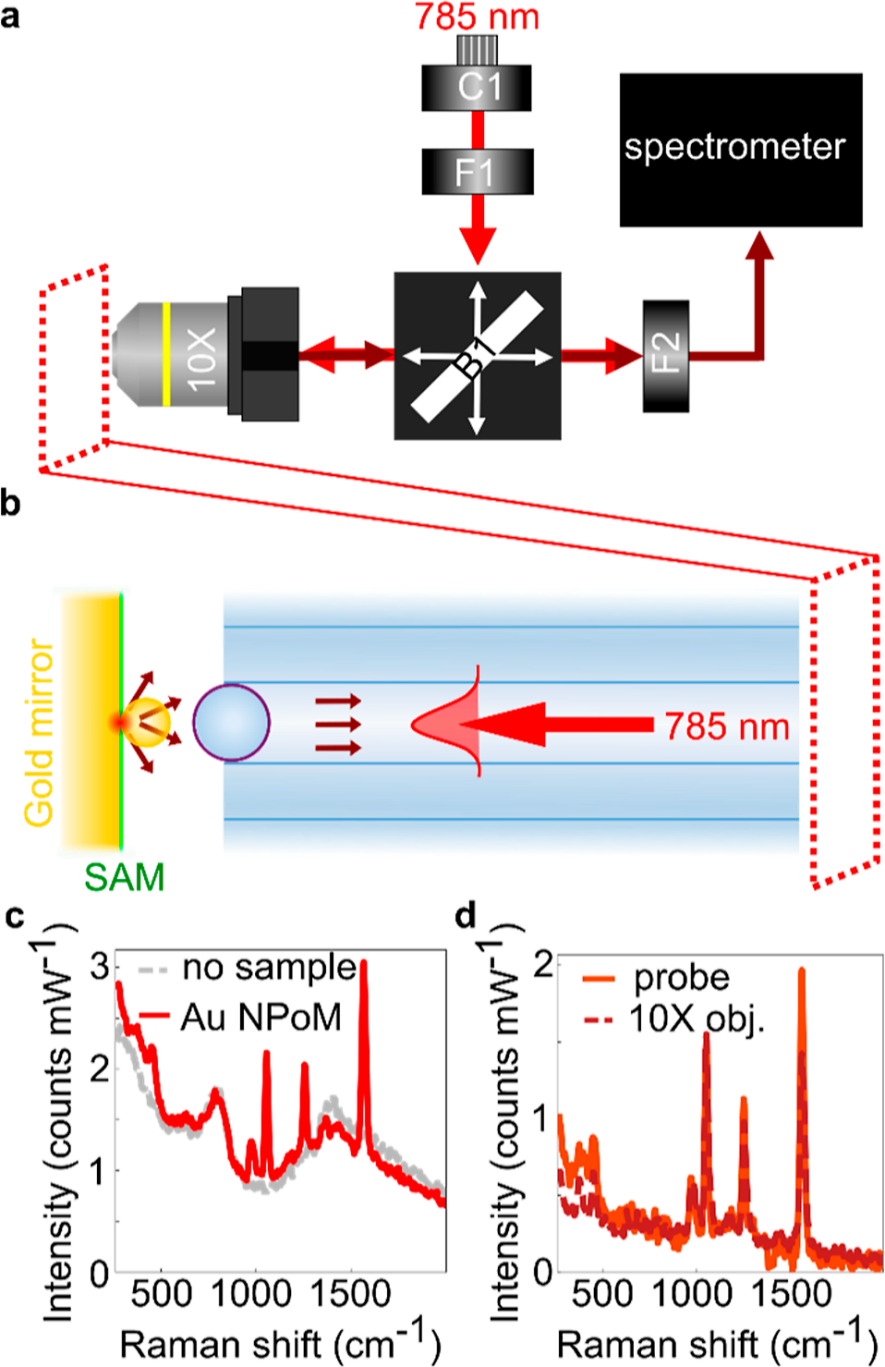
Raman measurements using the fiber-probe. (a) Schematic of the optical setup. 785 nm continuous laser light is delivered by a fiber-coupled collimator C1 and filtered by a bandpass filter F1. The generated backscattered Raman transmits through a 50:50 beam splitter B1, a notch filter F2, and is analyzed by the spectrometer. (b) Schematic of the probe and sample, 80 nm Au nanoparticle-on-mirror (NPoM) containing a BPT self-assembled monolayer. (c) Comparison of the Raman signal from NPoM with the background from the fiber probe. (d) Comparison of background-subtracted signals using the probe and microscope objective.

The next characterization now combines excitation and collection in measuring spontaneous backscattered Raman scattering (Figure 7a). A test sample consisting of 80 nm gold nanoparticles deposited on a mirror separated by a biphenyl-4-thiol (BPT) self-assembled molecular monolayer is used (for fabrication details, see Figure S4). This Au NPoM sample is a well-documented Raman sample with a strong signal-to-noise ratio, making it a good test sample. Its significant characteristics are an intense electric field hotspot between each nanoparticle and the mirror and a relatively wide emission angle of 60°.

A 10× objective was used to inject the filtered 785 nm laser light, Figure 7a, and to collect the Raman scattered light from the spectrometer. A notch filter was used to filter the pump light. A translation stage is used to move the NPoM sample to an optimal position. At the point of greatest signal intensity, spectra are integrated over 10 s. A third-order Savitzky–Golay filter smooths each spectrum. Raman scattering from the fiber–microlens-probe with and without a sample is compared in Figure 7b. Some background Raman scattering emerges from the microlens, including a peak around 800 cm^–1^ and a broad shoulder of about 1300 cm^–1^. The sharp Raman peaks of the ∼100 BPT molecules in the NPoM hotspot are clearly differentiated from the probe background.

The emission angle of the sample and the cone of collection of the probe are quite different. With NA = 0.12, the angular acceptance of the probe is 14°. Optimization of the microlens parameters to better match emission angles from each specific Raman sample would yield improved signal-to-noise.

To compare the probe effectiveness with a standard microscope objective, the NPoM Raman signal is also collected using a 10× objective (Figure 7c). Before smoothing, both data sets are background-subtracted. The fiber–microlens probe performance is comparable to the 10× objective, even without optimization of the microlens choice. This shows the promise of such probes for in situ measurements, where optical access with a conventional microscope is restricted.

#### Ex Situ Raman on Battery-Relevant Samples

**Figure 8:**
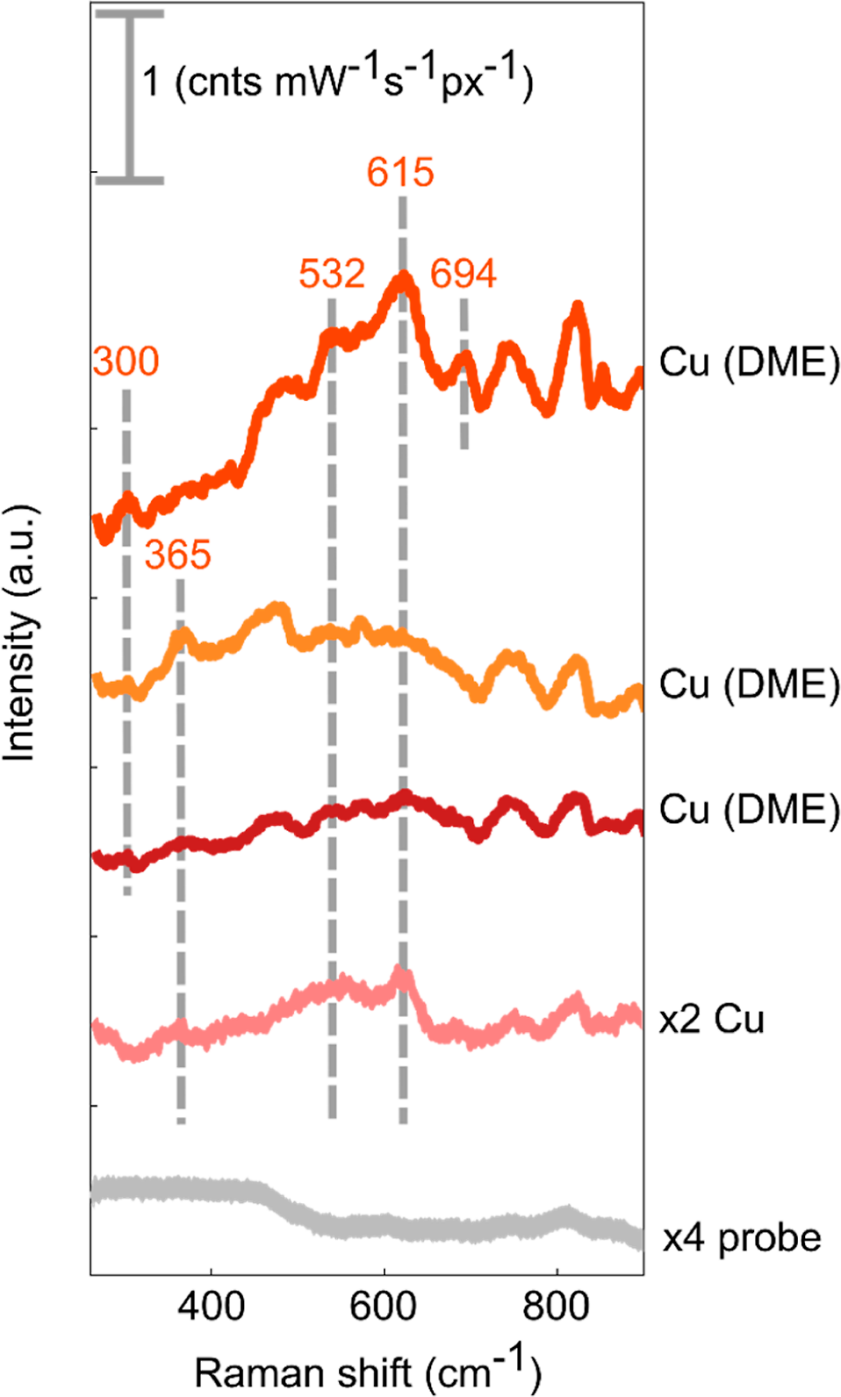
Ex situ Raman spectra taken using the fiber probe of two cycled copper electrodes. One electrode was washed with DME and dried, while the other was left to dry unwashed. The lower curve shows the Raman background of the probe itself.

To demonstrate the efficiency of the probe for Raman sensing on battery-electrodes, we have performed proof-of-principle experiments on a Cu electrode extracted from a cycled Li-ion battery. The Raman spectra from the copper electrode, cycled in LP57 electrolyte, were measured in air. Copper electrodes are used in anode-free lithium-battery chemistry.^57^ The same optical setup described in Figure 7a was used to obtain Raman spectra, moving the electrode sample relative to the probe to measure different regions. As with gold, copper is a plasmonic material that is appropriate for surface enhancement. Figure 8 shows spectra from different positions across a Cu electrode (washed with DME and left in an argon environment until measurement), a reference spectrum from an unwashed Cu electrode (intensity ×2), and a spectrum when no sample is present (intensity ×4). In some regions of the washed electrode, the Raman signal is enhanced by surface plasmons. As expected, the probe is very sensitive to its *z*-position relative to the sample, and we observed a range of only a few micrometers between no signal and maximum signal.

Different positions across the electrode show Raman bands indicative of different copper oxide species, which have been previously observed in in situ NMR studies of anode-free batteries.^57^ The Raman bands at 530 and 612 cm^–1^ are reported to be Cu_2_O and are present in all copper spectra.^58,59^ The band at 300 cm^–1^ is likely to be CuO.^59^ There are peaks at 815 and 750 cm^–1^ that are present in all Cu samples, which are likely electrolyte salts. This data thus shows the heterogeneity of the Raman spectra between different surface regions of the Cu electrode. This ex situ study demonstrates the promise of such a fiber probe for future in situ studies of battery electrode surfaces to monitor the evolution of the Cu surface during cycling.

## Conclusions and Outlook

To conclude, we prepared and characterized an integrated fiber–microlens probe for in situ and operando Raman spectroscopy. A 28 μm barium titanate glass microlens is embedded into a HCF. This probe design unifies excitation and collection within the hollow core of a fiber with a relatively simple design, which allows for a thin, flexible fiber–microlens probe with few fabrication stages. This method can be used on any combination of fiber and sphere to adapt to specific applications, such as lithium-ion battery monitoring.

Excitation and collection are characterized separately, for the first time to the best of our knowledge, using optical scanning microscopy and spectral mapping of a small-source photoluminescent sample. The characterization of the excitation intensity profile reveals that the microspheres used likely have an inhomogeneous refractive index. Further work needs to be carried out to investigate more homogeneous microspheres made of different materials (in progress).

By comparing the fiber probe collection efficiency for sample positions (*x*, *y*, *z*) with the intensity profile of the excitation pump light, two key conclusions can be drawn. First, the maximum collection and excitation efficiency points are so far inside the microlens. Second, the volume of effective collection is larger than that of effective excitation. We hypothesize that this is because the transmission of the collected signal involves extra higher-order fiber modes. This second conclusion suggests that the shape and size of the excitation profile currently have a larger influence on the intensity of backscattered Raman than the collection volume.

The effectiveness of this combination of fiber and microlens as a Raman probe is verified. The probe is found to achieve signals on the same order of magnitude as a 10× objective, despite being much smaller and minimally invasive. Comparing Raman intensities demonstrates the suitability of this fiber microlens as a viable alternative to a Raman microscope when optical access is restricted. Additional questions to resolve are how NA matching the sample and probe could further increase the signal-to-noise ratio by tuning the microlens refractive index.

The probe was found to be sensitive to surface changes across a cycled battery electrode, as demonstrated with ex situ Raman measurements of copper electrodes. This Raman probe is a promising in situ and operando Raman spectroscopy tool, particularly for monitoring electrode surface change inside sealed lithium-ion batteries. Further work to seal the probe without compromising the optical power of the microlens must be explored. This will allow for in situ battery-relevant samples to be explored. Existing examples of pouch cells with incorporated fiber probes greatly aid in this.^32^

## Methods

### Materials

The NANF was obtained from the University of Southampton ORC. Further characterization can be found in previous work.^29^ The single-mode optical fiber was S405-XP, fwhm 3.8 μm, outer diameter 125 μm cladding from Thorlabs. The microspheres used were barium titanate solid glass microspheres 27–32 μm from Cospheric. MPLN 4× Olympus 0.10 NA, MPLN 10× Olympus 0.25 NA, MPLFLN 10× Olympus 0.3 NA, and 60× 0.85 NA microscope objectives were used.

### Chemicals

CdTe core-type QDs with fluorescence at 770 nm, which were COOH functionalized (777994 powder), (3-aminopropyl)triethoxysilane (APTES, 440140), and hydrochloric acid (25148), were all obtained from Sigma-Aldrich, and methanol (10284580) was obtained from Fisher Scientific.

### QD Deposition

This was performed following a previously reported protocol, with slight modifications.^55^ In this method, APTES is used as an anchoring agent to attach QDs to the glass substrate. APTES forms regular and stable bonds to the silica glass, after which the COOH-functionalized QDs can electrostatically interact with the APTES, forming a dense, homogeneous, and stable two-dimensional monolayer.

A freshly cleaved single-mode fiber is immersed in a solution of HCl in MeOH (1:1) for 30 min. The fiber is then left in 5% APTES in EtOH for 1 h before being sonicated in MeOH, rinsed with IPA, and dried under nitrogen. The fiber is then left in a 1 mg mL^–1^ solution of CdTe core–shell types QDs in H_2_O for 8 h. Finally, it is rinsed in Milli-Q H_2_O and dried under nitrogen flow.

### Electrode Sample Preparation

Copper foil was cut into 16 mm discs and submerged in concentrated acetic acid for 10 min to remove the surface oxidation layer. Acetic acid was removed by flowing dry nitrogen over the surface, and then the AcH-Cu discs were dried at 100 °C under a vacuum overnight before being stored under argon. The copper discs were run in a linear sweep voltammetry with LP57 for 1 sweep (sweep rate = 5 mV/s) in a 3-electrode cell assembled under argon (TSC Surface by rhd instruments) after a 48 h rest. After the sweep, the cell was disassembled under argon and washed with DME. The samples were roughly sealed in argon until use.

### Instrumentation

Transmission and photoluminescence data are collected using a fiber-coupled Ocean Optics QE65000 spectrometer. 785 nm light is delivered through a fiber-coupled continuous-wave Ti:sapphire laser (MSquared SolsTis). Transmission data is collected using a broadband fiber-coupled supercontinuum laser (NKT Photonics SuperK Compact). 405 nm light is delivered from a diode laser (Thorlabs CPS405). A closed loop 3-Axis NanoMax stage with differential drives is used for scans and mapping.

### Wave Model

A two-dimensional model is constructed and simulated using a Lumerical finite-difference time-domain solver. The input field is taken to be the fundamental mode of the NANF, which is calculated using the Lumerical MODE package. For a HCF diameter of 28 μm, the fundamental eigenmode is found to be well-approximated by a Gaussian with a fwhm of 16.4 μm. The sphere used in the model has a diameter of 28 μm (2sf) and a refractive index of 1.9.

## Data Availability

The data that support the findings of this study are available from the corresponding author, T.G.E., upon reasonable request.

## Supporting Information Available

Additional information on fiber internal structure; modeled and measured fiber transmission properties; and detailed setup descriptions for characterizing signal collection (PDF)
