# Supplementary material for: Microlens Hollow-Core Fiber Probes for Operando Raman Spectroscopy
Source: ACS Photonics. 2024 Jul 22;11(8):3167–77. doi: 10.1021/acsphotonics.4c00525 (PMC11342360; doi:10.1021/acsphotonics.4c00525)
Supplement: Supplementary file 1 — ph4c00525_si_001.pdf [file ph4c00525_si_001.pdf]

Supporting Information

Microlens hollow-core fibre probes for operando Raman spectroscopy

Megan J. Groom,[1,2] Ermanno Miele,[1,2] Jonathan Pinnell,[1] Matthew G. Ellis,[1] Jessica B. McConnell,[2,3] Hesham Sakr,[4,5] Gregory Jasion,[4] Ian Davidson,[4] Natalie Wheeler,[4] Yongmin Jung,[4] Francesco Poletti,[4] Svetlana Menkin,[2,3] Marlous Kamp,[1,6] Jeremy J. Baumberg,[1] and Tijmen G. Euser[1,2,*]

1. Nanophotonics Centre, Department of Physics, Cavendish Laboratory, University of Cambridge, CB3 0HE, Cambridge, U.K.

2. The Faraday Institution, Quad One, Harwell Science and Innovation Campus, Didcot OX11 0RA, Oxford, U.K.

3. Yusuf Hamid Department of Chemistry, University of Cambridge, CB2 1EW, Cambridge, U.K.

4. Optoelectronics Research Centre, University of Southampton, SO17 1BJ, Southampton, U.K.

5. now with Microsoft Azure Fibre, SO51 9DL, Romsey, U.K.

6. Van 't Hoff Laboratory for Physical & Colloid Chemistry, Department of Chemistry, Utrecht University, 3584 CH Utrecht, The Netherlands

Total number of:

Pages	4
Figures	4
Tables	1

Optical transmission of the NANF probe

Figure S1 and Table S1 show the properties of the internal structure of the NANF used in this work, Fibre B from Sakr *et al.*. Figure S2 shows the ARROW model predictions for this fibre, given its strut thickness, clearly showing its suitability for 785 nm Raman applications. Figure S3 shows the actual attenuation data for the fibre, as seen in Sakr *et al.*. [29] At 10m in length, the resonance window of the fibre is measured to be 645-1021 nm. Hence, this fibre guides from the pump wavelength of 785 nm up to Raman shifts of 2945 cm^{-1}, covering the Raman fingerprint region.

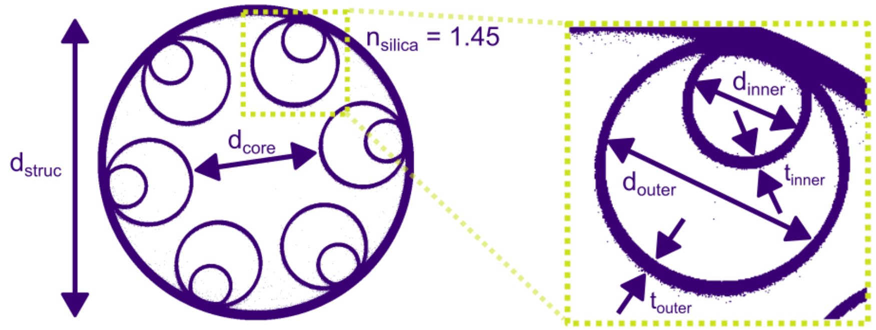

Figure S1 An illustration of the parameters of fibre internal structure, using an SEM image to derive the glass structure outline.

Table SI 1 The parameters of the fibre internal structures, as shown in Figure S1.

**Parameter**	**Symbol**	**Value**
Wavelength	λ	785 nm
Refractive index of hollow region	n_{hollow}	1.00
Refractive index of glass	$n_{sillica}$	1.45
Diameter of internal structure	d_{struc}	65 μm
Diameter of core	d_{core}	28.3 μm
Diameter of outer capillaries	d_{outer}	18.75 μm
Diameter of inner capillaries	d_{inner}	9.40 μm
Thickness of outer capillaries	t_{outer}	580 nm
Thickness of inner capillaries	t_{inner}	580 nm

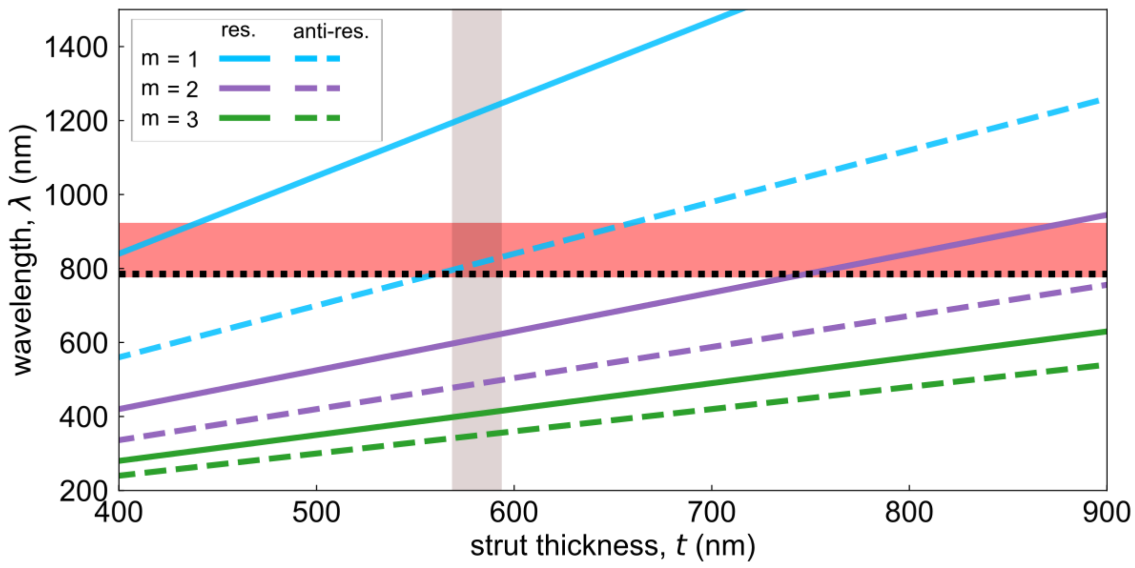

Figure S2 ARROW model predictions for the NANF with strut thickness 580 ±10 nm. 785 nm pump wavelength for Raman spectroscopy is shown as a black horizontal line, and relevant Raman scattering wavelengths are shown as a red shaded region (0-1900 cm^{-1}).

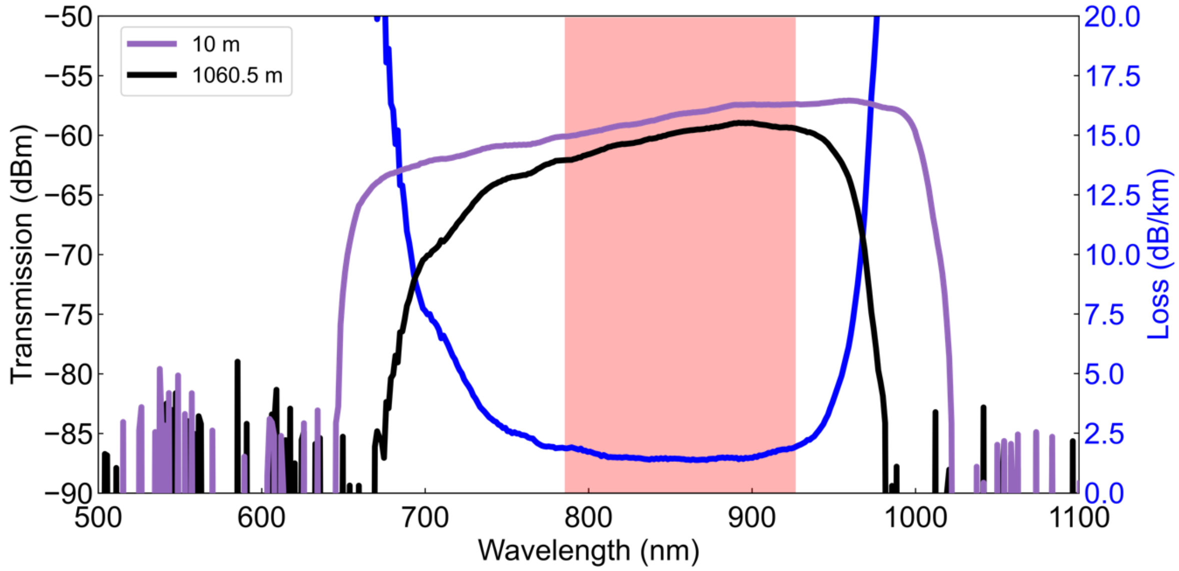

Figure S3 The measured transmission and loss of the NANF used, as seen in Sakr et al.. (29) The red shaded region corresponds to wavenumbers in the Raman fingerprint region 0-1900 cm^{-1}.

Characterizing signal collection

Figure S4 shows the optical setup for the collection characterisation experiments. For Figure 5 and 6, the excitation set-up is the same and shown in Figure S4a. The 405 nm laser diode is filtered by a 405 nm bandpass filter and launched into single mode fibre with a 4× objective, by placing the SMF on a translation stage.

To collect the signals shown in Figures 5 and 6, a 250 mm focal length lens launched light into a MMF. This 200 μm MMF delivers light to a fibre-coupled Ocean Optics spectrometer, as shown by Figure S4 b – d. When collecting the 780 nm QD emissions, as in Figure 5 d and Figure 6 b, 450 nm and 650 nm long pass filters are used.

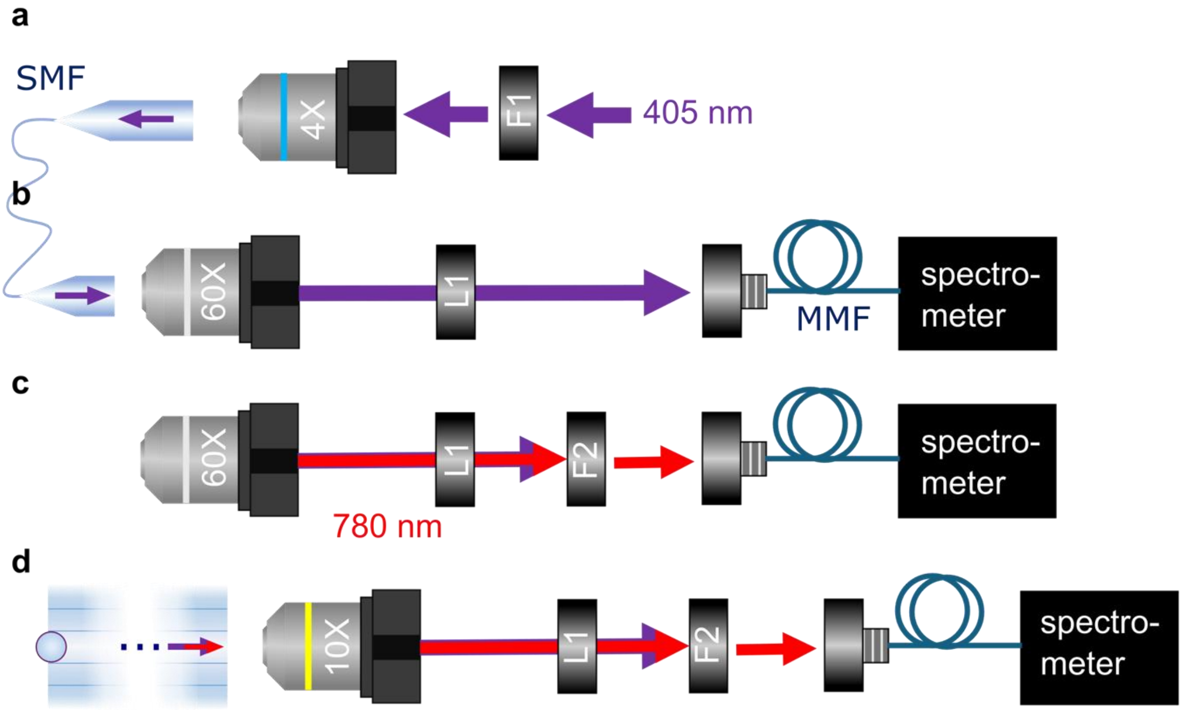

Figure S4 Optical set-up variations for characterising the signal collection of the probe. (a) The 405 nm laser diode is filter by a 405 nm bandpass, F1, and coupled into the unmodified SMF facet with a 4× objective. Depending on the sample, (b) uncoated SMF, (c) a SMF coated with QDs, or (d) the signal of the coated SMF collected by the fibre-probe, a 60× or 10× objective collects the transmitted signal. The signal is focused into a MMF by lens L1 with focal length 250 mm. The MMF delivers the light to the fibre-coupled spectrometer. The QD emission signal is filtered by F2, 650 nm long pass filter, to remove residual 405 excitation.

Preparation of NPoM samples

The Au manoparticle on a mirror (NPoM) samples (discussed in Figure 7), were fabricated following the procedure outlined in Ref [S1].

References

(S1) de Nijs, B.; Benz, F.; Barrow, S.J. *et al.* Plasmonic tunnel junctions for single-molecule redox chemistry. *Nat. Commun.* **2017**, *8*, 994.
